# Six-month safety evaluation of robenacoxib tablets (Onsior™) in dogs after daily oral administrations

**DOI:** 10.1186/s12917-018-1566-1

**Published:** 2018-08-17

**Authors:** Céline E. Toutain, Patrick Brossard, Stephen B. King, Rainer Helbig

**Affiliations:** 1 Elanco Animal Health, a Division of Eli Lilly and Company, Mattenstrasse 24a, CH-4058 Basel, Switzerland; 2 Elanco Animal Health, a Division of Eli Lilly and Company, 2500 Innovation Way, Greenfield, IN 64140 USA

**Keywords:** Robenacoxib, Onsior™, Tablet, Target animal safety, NSAID, Dog

## Abstract

**Background:**

Robenacoxib is a non-steroidal anti-inflammatory drug available for canine and feline use for the control of pain and inflammation marketed as Onsior™. The aim of this target animal safety study was to evaluate the 6-month safety profile of oral robenacoxib administration. It was a randomized, negative-controlled, parallel group study. Thirty-two healthy, young, experimentally naïve, purebred Beagle dogs were administered 0 (sham control, Group 1), 2, 6, and 10 mg/kg robenacoxib (corresponding to the upper end of the dosage range [1X, Group 2] and multiples thereof [3X and 5X, Group 3 and 4]), orally once daily for 6 months. Assessment of safety included general health and clinical observations, physical, neurological, ophthalmological and electrocardiographic examinations, gross and histopathological examinations and clinical pathology evaluations. Blood samples were collected for toxicokinetic assessment of robenacoxib.

**Results:**

No serious adverse events were reported. When compared with control, no treatment effect was observed for body weight, feed or water consumption, clinical pathology, urinalysis and fecal examination parameters. There were no treatment-related changes in stifle joint tissues and microscopic/histopathology examinations of all tissues/organs were normal. Salivation and soft feces were noted in all groups but observed more frequently in the treated groups as compared with control. On Day 178, increased buccal mucosal bleeding times were observed in two treated animals (Group 3 and 4) and one dog in Group 4 displayed a retinal change. Decreased hopping and conscious proprioception was noted in four treated dogs. One dog in Group 2 had ventricular premature complexes. Post-mortem changes included mild, red foci on the cecum in one dog (Group 3) and minimal duodenal discoloration in one dog (Group 4), with no corresponding histological findings in either dog. Ovarian weights were decreased in females from Group 3 and 4 with no gross or histological changes in the ovaries. Blood concentrations of robenacoxib confirmed systemic exposure of treated dogs. Exposure increased with increasing doses and there were no accumulation of robenacoxib in blood.

**Conclusions:**

Robenacoxib was well tolerated at doses from 2 to 10 mg/kg/day and this 6-month study supports the safe use of Onsior™ (robenacoxib) tablets in dogs for the intended dosing regimen.

## Background

The non-steroidal anti-inflammatory (NSAID) drug robenacoxib has been developed for veterinary use in cats and dogs [1, 2]. It is highly selective for inhibition of the cyclooxygenase (COX)-2 [3–5] with a fast onset of action in cats [6] and dogs [7] and an improved safety profile as compared with non-selective NSAIDs [8–10]. It is marketed as Onsior™ and available in many countries as both injection and oral formulations for both species [11]. The oral formulation for dogs (Onsior™ tablets, Elanco Animal Health) was approved in Europe since 2008 [11] for the treatment of pain and inflammation associated with chronic osteoarthritis in dogs at a dose of 1 mg/kg (with a range 1–2 mg/kg) and more recently in the United States in 2016 [12] for the control of postoperative pain and inflammation associated with soft tissue surgery at a dose of 2 mg/kg (with a range 2–4 mg/kg).

Previous studies have demonstrated an excellent safety profile for robenacoxib when administered orally to healthy young beagle dogs at daily doses of up to 40 mg/kg for 1 month and up to 10 mg/kg for 6 months [8]. These studies were conducted with a prototype formulation of robenacoxib which necessitated a target animal safety study to be conducted with the final commercial product in order to obtain global marketing authorizations, as outlined in the guideline for evaluating the target animal safety of new veterinary products (VICH Guideline 43) [13].

The objective of the present study was therefore to investigate the long-term safety of the veterinary medicinal product (Onsior™ tablets) administered orally to dogs once daily at dosages of 0, 2, 6 and 10 mg/kg for a total duration of six consecutive months.

## Methods

### Objective and standards

The objective of this study was to evaluate the target animal safety of robenacoxib tablets (Onsior™, Elanco Animal Health) when administered daily to young adult dogs at 0, 2, 6 and 10 mg/kg for 6 months.

This nonclinical study was conducted in accordance with Good Laboratory Practice (GLP) [14, 15] and according to the guideline for evaluating the target animal safety of new veterinary products (VICH Guideline 43) [13]. The study was reviewed and approved by the study site Institutional Animal Care and Use Committee. It was in compliance with the U.S. Department of Agriculture’s (USDA) Animal Welfare Act (9 CFR Parts 1, 2 and 3) and followed The Guide for the Care and Use of Laboratory Animals [16, 17]. This manuscript was prepared in compliance with the ARRIVE guidelines for reporting animal in vivo experiments [18].

### Animals and maintenance

A total of 40 (20 males and 20 females; approximately 5 months of age) healthy, experimentally naïve, vaccinated Beagle dogs were obtained commercially from a laboratory animal supplier. All animals were acclimatized for 50 days before study initiation. Following clinical pathology evaluations and a detailed physical and neurological examination before study start, 32 animals (16 males and 16 females) were considered suitable for the study and enrolled.

All dogs were housed individually in approximately 1.2 sq.m runs on raised floor caging that provided adequate room for exercise and under climate controlled conditions (temperature, 18 to 29 °C; humidity, 30% to 70%). Fluorescent lighting was provided for approximately 12 h per day. Animals were fed ad libitum with a certified commercial canine diet for approximately 6 h daily and water was available ad libitum.

### Allocation to groups

This was a randomized, controlled, blinded, 6-month study with a parallel design. Using a block randomization procedure stratified by sex and weight, 32 animals (16 males weighing 8.28 to 13.79 kg and 16 females weighing 6.61 to 9.20 kg) were randomized on Day − 1 to one of the following four groups (4 males and 4 females per group): Group 1, placebo (sham); Group 2, 2 mg/kg robenacoxib; Group 3, 6 mg/kg robenacoxib; and Group 4, 10 mg/kg robenacoxib.

The animals were identified by a microchip implant and vendor tattoo. The cage number of the animal was identified by the study number, animal number, and sex, but did not include the treatment to blind personnel responsible for any data collection.

### Investigational veterinary product and administration

The investigational veterinary products (i.e. the pharmaceutical form being evaluated) were supplied as commercial Onsior™ tablets containing 5, 10, 20 or 40 mg of robenacoxib. The number and size of the tablets administered orally depended on the most recent body weights and the target dose level required (either 2, 6 or 10 mg/kg). The control animals were sham dosed. Tablets were administered after overnight fasting once daily for a total of 26 weeks, from Day 1 to 181 (the day before study completion). After each dose, approximately 5 mL tap water was administered orally with a syringe. Feed was offered 1 to 2 h following dosing. The tablets were administered without feed in order to maximize systemic exposure since robenacoxib oral bioavailabilty is decreased with co-administration with feed [19].

### Measurements and variables recorded

Throughout the study, all animals were observed at least twice daily for general health, morbidity, mortality, injury, and availability of feed and water. On days of treatment, detailed clinical observations were conducted twice per day, at least 6 h apart, once in the morning at 1 h post-dose (±15 min) and once in the evening. The observations included, but were not limited to, eyes, mucus membranes, respiratory system, circulatory system, autonomic and central nervous systems, somatomotor activity and behavior pattern.

Body weights were measured at least weekly throughout the study. Feed and water consumption were measured daily. A veterinarian conducted physical and neurological examinations 2 days before and 7, 30, 91, 150, and 178 days after the first dose. General physical examinations included general condition and behavior, general ocular without ophthalmoscope, integument, musculoskeletal, gastrointestinal, body temperature, cardiovascular and respiratory including assessment by auscultation, and reproductive system by external examination.

Electrocardiography and ophthalmoscopic examinations were conducted once during acclimatization and within 1 week before the scheduled study completion. Evaluations were performed by a board-certified veterinary cardiologist and ophthalmologist, respectively.

The complete panel of clinical pathology endpoints (hematology, blood chemistry and urinalysis) according to VICH GL43 [13] was analyzed during acclimatization and 30, 91, and 181 days after the first dose. Buccal mucosal bleeding time (BMBT) was evaluated during acclimatization and 31, 92, and 178 days after the first dose.

Fecal and urine samples were collected for analysis once during acclimatization and 30, 91, and 181 days after the first dose. Feces were weighed and were then observed for color, consistency, size, parasites, and other abnormalities.

### Blood samples for toxicokinetics

Blood samples (2 mL) were collected from all animals via the jugular vein for determination of the blood concentrations of robenacoxib. Samples were collected pre-dose and at 15 and 30 min, 1, 2, 5, 8, 12, and 24 h post-dosing on the day of the first dose and 30 and 150 days after the first dose. Samples were placed in tubes containing K_2_ EDTA and stored frozen until analyzed.

Determination of robenacoxib blood concentrations has been described previously [19]. Briefly, following an initial solid phase extraction, robenacoxib was quantified by high pressure liquid chromatography – ultra violet (HPLC-UV) for concentrations > 500 ng/mL and by liquid chromatography – mass spectrometry (LC-MS) for concentrations < 500 ng/mL. The analytical method was validated using quality control spiked matrix specimens run with each sequence of unknown samples, and independent of calibration standards. The lower limit of quantitation (LLOQ) of the analytical method was 2 ng/mL.

The analytical data were processed as individual time versus blood concentration profiles using the validated pharmacokinetic evaluation software DEBA (Data Evaluation in BioAnalytics; release 4.0, Copyright Dr. C.N. Thumm-Kraus, Software und Beratung, 1999) and the following parameters were estimated: AUClast, Tmax and Cmax.

### Pathological examination

Euthanasia was performed by sodium pentobarbital solution administration. A board-certified veterinary pathologist conducted complete post-mortem examinations on all animals at the necropsy 182 days after the first dose. Macroscopic examinations were conducted for external or internal abnormalities. A full complement of organs and tissues, including tibial cartilage, was collected from all animals and fixed in neutral buffered formalin. Tibia specimens were placed into 5% formic acid and decalcified for 5 weeks prior to histological processing.

Body and organ weights were recorded for all animals and organ weight ratios were calculated (relative to body and brain weights). Histopathology examination of fixed hematoxylin and eosin-stained paraffin sections was performed by a blinded board-certified veterinary pathologist and any observed lesions were graded using a 4-step grading system (minimal, mild, moderate and severe) to enable comparison between the dose groups.

### Statistical analysis of data

Statistical analyses were conducted using SAS® (Statistical analysis system, Version 8.2, Cary, North Carolina: SAS Institute Inc.). For each endpoint, data from the treatment groups (Groups 2, 3, or 4) were compared with data from the control group (Group 1). The experimental unit was the individual animal.

Endpoints measured one time post-treatment that did not include a pre-treatment measurement (absolute organ weights and organ weight relative to body and brain weights) were analyzed using an analysis of variance (ANOVA) with classification variables ‘treatment’, ‘sex’, and the two-way interaction term ‘treatment by sex’. The treatment main effect was evaluated only if the ‘treatment by sex’ was not significant (*p* > 0.05). If the treatment effect was significant (*p* ≤ 0.10), pair-wise comparisons of treatment groups with the control group were tested.

Endpoints measured multiple times post-treatment that included a pre-treatment measurement (body weights, feed consumption, and clinical pathology) were analyzed using repeated measures analysis of covariance (RMANCOVA) with classification variables ‘treatment’, ‘time’, and ‘sex’; the two-way interactions ‘treatment by time’, ‘treatment by sex’, and ‘sex by time’; the three-way interaction ‘treatment by time by sex’; and the pre-treatment value closest to dosing was used as a covariate. If the ‘treatment by time’ interaction was significant (*p* ≤ 0.10), each treatment group was compared to the control through the simple effect of ‘treatment’ for each time point. The treatment main effect was evaluated only if all the two-way and three-way interactions were not significant. If the treatment effect was significant (p ≤ 0.10), pair-wise comparisons of treatment groups with the control group were tested.

## Results

### Investigational veterinary product doses

The actual mean dose levels for males and females for the groups receiving 2, 6 and 10 mg/kg of robenacoxib was 2.24, 6.26, and 10.24 mg/kg/day, respectively. Individual robenacoxib doses reached or exceeded the intended oral dose levels in all treatment groups.

### Clinical assessments

There was no mortality and no serious adverse events in any animal until scheduled termination on Day 182. All dogs were healthy through study termination. Dogs gained approximately 20% weight during the study. Body weights and feed consumption changes were similar between controls and treated groups throughout the study (Table 1). There were no statistically significant differences between control and treated animals (*P* = 0.4147) for feed consumption (Table 1). No relevant variations in water consumption were observed.

**Table 1:** Summary of body weight and feed consumption; mean (SD), *N* = 8

Variable (units)		Group 1 (sham)	Group 2 (2 mg/kg)	Group 3 (6 mg/kg)	Group 4 (10 mg/kg)	*P*-value
						Treatment
Body weight (kg)	Pretest	9.32 (2.17)	9.27 (2.00)	9.18 (1.52)	9.12 (1.51)	0.2736
	Week 26	11.24 (2.40)	11.29 (2.25)	11.19 (1.71)	10.84 (1.59)	
Feed consumption (g/animal/day)	Pretest	230.3 (44.2)	227.1 (36.8)	211.9 (45.4)	238.7 (41.9)	0.4147
	Week 26	283.5 (66.2)	311.9 (40.4)	277.7 (54.2)	360.8 (224.7)	

During animal observations, salivation was noted in all groups, but most frequently in Group 3 (6 mg/kg). Similarly soft/mucoid/watery feces were noted in all groups, but more frequently in the treated groups as compared to control group. On Day 178, unilateral or bilateral decreased hopping was noted in three treated animals (two dogs in Group 2; 2 mg/kg and one dog in Group 4; 10 mg/kg). One of these dogs (Group 2) had abnormalities noted on neurological examination (absence of panniculus reflex) prior to treatment. Unilateral decreased conscious proprioception was also noted on Day 178 in one treated dog in Group 4.

Increased BMBTs were observed in one female (Group 3; 6 mg/kg) and one male (Group 4; 10 mg/kg) on Day 178 (5 min 55 s and 5 min 10 s, respectively). Both dogs had values within normal limits (i.e. between 1 to 5 min) prior to treatment (1 min 55 s and 3 min 51 s, respectively). Mean BMBT values were similar between the control and the treated groups.

One female (Group 4; 10 mg/kg) had a single retinal fold in the tapetal fundus of the left eye noted on Day 177.

Cardiovascular auscultation was normal in all dogs. Three ventricular premature complexes (VPCs) were noted in the terminal ECG on Day 178 of one male (Group 2; 2 mg/kg).

Examination of the reproductive system was normal in all dogs. Urinalysis parameters and fecal analysis findings were comparable between control and treated groups at all time points.

### Clinical pathology

Summary data for selected hematology and clinical chemistry parameters are presented in Tables 2 and 3. The hematology values were in general comparable between control and treated animals with a few exceptions. A treatment by time effect was identified for hemoglobin (increased in Group 2 on Day 91; *P* = 0.0283) and a treatment effect on reticulocytes (increased in Group 3; *P* = 0.0215) and eosinophils (increased in Group 4; *P* = 0.0003).

**Table 2:** Summary of hematology data; mean (SD), N = 8

Variable (reference range and units)^a^	Day	Group 1 (sham)	Group 2 (2 mg/kg)	Group 3 (6 mg/kg)	Group 4 (10 mg/kg)	P-value^2^
						Treatment
White blood cell count (5.0–14.1 × 10^3^/μL)	Pretest	12.25 (2.10)	12.81 (2.53)	15.81 (5.14)	13.88 (4.39)	0.2703
	30	9.91 (0.95)	12.29 (2.72)	11.74 (2.51)	11.31 (1.71)	
	91	10.15 (1.80)	10.45 (1.36)	12.83 (2.69)	12.46 (3.96)	
	181	9.34 (2.25)	11.03 (4.63)	11.30 (1.81)	10.59 (1.99)	
Red blood cell count (4.95–7.87 × 10^6^/μL)	Pretest	7.079 (0.396)	7.344 (0.407)	6.994 (0.582)	6.935 (0.597)	0.7588
	30	7.124 (0.570)	7.169 (0.677)	6.940 (0.492)	6.910 (0.357)	
	91	7.103 (0.411)	7.590 (0.701)	6.995 (0.448)	6.984 (0.534)	
	181	6.835 (0.380)	6.880 (0.538)	6.633 (0.389)	6.948 (0.289)	
Reticulocytes (< 80 × 10^3^/μL)	Pretest	42.70 (14.25)	51.36 (35.09)	45.20 (13.76)	42.90 (13.09)	**0.0962**
	30	32.89 (14.74)	40.03 (16.68)	47.28 (16.49)	35.43 (12.29)	
	91	13.73 (8.39)	22.65 (9.31)	26.43 (15.38)	22.76 (8.48)	
	181	28.48 (24.78)	21.51 (8.90)	41.43 (18.70)	30.53 (15.87)	
Eosinophils (0.0–1.3 × 10^3^/μL)	Pretest	0.226 (0.062)	0.278 (0.179)	0.270 (0.097)	0.358 (0.102)	**0.0005**
	30	0.248 (0.122)	0.265 (0.137)	0.291 (0.095)	0.453 (0.141)	
	91	0.228 (0.057)	0.274 (0.115)	0.288 (0.089)	0.465 (0.207)	
	181	0.276 (0.073)	0.301 (0.112)	0.340 (0.109)	0.598 (0.176)	
Platelets (211–621 × 10^3^/μL)	Pretest	333.5 (52.7)	311.9 (49.7)	269.3 (63.1)	300.3 (46.4)	0.8921
	30	346.8 (81.4)	333.9 (36.1)	295.9 (90.0)	315.8 (37.3)	
	91	313.9 (62.7)	313.3 (61.6)	301.5 (68.4)	306.4 (54.4)	
	181	301.3 (84.0)	298.6 (63.8)	269.0 (90.2)	277.1 (41.5)	
Hemoglobin (11.9–18.9 g/dL)	Pretest	15.54 (0.73)	15.70 (0.85)	15.49 (1.28)	15.04 (1.30)	0.8821
	30	16.09 (1.00)	15.90 (1.02)	15.88 (1.13)	15.49 (1.20)	
	91	15.60 (0.66)	16.68 (1.35)	15.69 (1.00)	15.45 (1.26)	
	181	15.76 (0.79)	15.53 (1.21)	15.45 (0.92)	15.93 (0.61)	
Hematocrit (35–57%)	Pretest	43.94 (2.12)	44.86 (2.46)	43.63 (3.66)	42.56 (3.51)	0.8776
	30	45.11 (3.16)	44.45 (2.98)	44.31 (3.31)	43.20 (2.89)	
	91	44.05 (2.95)	46.68 (4.00)	43.66 (2.86)	42.63 (4.16)	
	181	42.65 (2.40)	42.53 (3.36)	41.71 (2.28)	43.19 (1.57)	

Footnote:

^a^ These reference ranges are to be used for information only and the pretest values collected before the dosing period during acclimatization should be used for interpretation in the context of this study

^2^ *P*-values in bold denote significance

**Table 3:** Summary of clinical chemistry data; mean (SD), N = 8

Variable (reference range and units)^a^	Day	Group 1 (sham)	Group 2 (2 mg/kg)	Group 3 (6 mg/kg)	Group 4 (10 mg/kg)	*P*-value^2^
						Treatment
Alkaline phosphatase (1–114 U/L)	Pretest	89.3 (25.9)	101.5 (21.9)	86.4 (11.5)	94.4 (16.1)	0.8687
	30	76.8 (28.7)	82.9 (16.8)	70.0 (10.6)	79.3 (15.0)	
	91	47.9 (17.6)	53.5 (14.2)	45.1 (10.9)	50.6 (8.5)	
	181	36.6 (17.1)	41.8 (15.3)	36.1 (6.0)	42.1 (8.6)	
Aspartate aminotransferase (28–60 U/L)	Pretest	35.1 (5.3)	35.5 (5.3)	38.1 (8.3)	36.1 (9.5)	0.7180
	30	31.1 (6.8)	30.3 (6.2)	30.1 (4.0)	30.4 (3.8)	
	91	30.4 (5.5)	34.8 (3.8)	31.3 (5.4)	26.8 (3.5)	
	181	27.9 (8.6)	27.1 (5.3)	27.8 (4.7)	28.8 (3.7)	
Alanine aminotransferase (10–109 U/L)	Pretest	27.6 (5.0)	28.8 (4.1)	28.4 (7.8)	28.6 (7.2)	0.8099
	30	30.6 (7.1)	29.4 (5.6)	32.6 (5.8)	29.3 (3.5)	
	91	30.5 (6.6)	36.0 (10.1)	31.9 (5.1)	34.1 (12.9)	
	181	32.3 (12.8)	29.0 (7.0)	33.6 (9.5)	37.6 (12.8)	
Creatine kinase (52–368 U/L)	Pretest	312.1 (153.0)	261.8 (98.7)	278.8 (52.5)	324.4 (228.5)	0.7749
	30	216.5 (57.9)	186.8 (56.5)	182.1 (26.6)	211.4 (40.8)	
	91	185.8 (38.8)	248.3 (153.8)	182.3 (44.3)	163.8 (41.6)	
	181	197.1 (81.3)	174.4 (55.3)	180.9 (59.0)	179.1 (45.8)	
Urea nitrogen (8–28 mg/dL)	Pretest	11.0 (2.6)	11.5 (1.6)	11.4 (2.1)	11.1 (1.9)	0.5554
	30	13.8 (2.8)	13.3 (1.6)	13.4 (2.3)	13.8 (1.8)	
	91	15.3 (1.9)	15.0 (2.8)	14.1 (2.9)	13.0 (2.4)	
	181	15.1 (2.4)	15.5 (2.5)	14.3 (2.8)	15.6 (1.8)	
Creatinine (0.5–1.7 mg/dL)	Pretest	0.59 (0.08)	0.60 (0.05)	0.59 (0.04)	0.58 (0.07)	0.8214
	30	0.70 (0.14)	0.69 (0.06)	0.66 (0.07)	0.68 (0.07)	
	91	0.73 (0.10)	0.74 (0.07)	0.69 (0.08)	0.69 (0.10)	
	181	0.76 (0.14)	0.74 (0.09)	0.74 (0.05)	0.74 (0.07)	
Total Protein (5.4–7.5 g/dL)	Pretest	5.83 (0.20)	5.56 (0.17)	5.75 (0.31)	5.68 (0.27)	0.6166
	30	5.90 (0.26)	5.61 (0.11)	5.76 (0.36)	5.75 (0.25)	
	91	5.69 (0.16)	5.46 (0.15)	5.55 (0.40)	5.60 (0.23)	
	181	6.46 (0.14)	6.19 (0.14)	6.23 (0.38)	6.25 (0.30)	
Albumin (2.9–3.9 g/dL)	Pretest	3.34 (0.16)	3.18 (0.12)	3.20 (0.15)	3.21 (0.10)	0.0631
	30	3.24 (0.17)	3.08 (0.15)	3.09 (0.16)	3.19 (0.16)	
	91	3.48 (0.15)	3.35 (0.12)	3.24 (0.19)	3.36 (0.11)	
	181	3.64 (0.12)	3.31 (0.12)	3.31 (0.22)	3.45 (0.13)	
Direct bilirubin (0.0–0.3 mg/dL)	Pretest	(−)	(−)	(−)	(−)	0.4098
	30	0.00 (0.00)	0.01 (0.04)	0.00 (0.00)	0.04 (0.05)	
	91	0.04 (0.05)	0.00 (0.00)	0.03 (0.05)	0.00 (0.00)	
	181	0.01 (0.04)	0.00 (0.00)	0.01 (0.04)	0.01 (0.04)	
Cholesterol (135–278 mg/dL)	Pretest	164.3 (30.1)	145.8 (24.3)	155.5 (22.5)	158.6 (23.1)	0.1893
	30	156.6 (26.6)	136.5 (19.2)	142.4 (14.7)	146.0 (18.0)	
	91	153.1 (41.3)	131.1 (18.6)	133.9 (12.1)	147.4 (24.8)	
	181	194.4 (54.2)	186.1 (55.0)	177.1 (37.6)	158.0 (30.4)	
Calcium (9.1–11.7 mg/dL)	Pretest	11.01 (0.33)	10.73 (0.21)	10.86 (0.26)	10.91 (0.26)	**0.0423**
	30	11.03 (0.28)	10.93 (0.18)	10.85 (0.26)	10.99 (0.27)	
	91	10.54 (0.15)	10.46 (0.09)	10.30 (0.20)	10.46 (0.31)	
	181	10.86 (0.37)	10.74 (0.11)	10.69 (0.18)	10.88 (0.23)	

Footnote:

^a^ These reference ranges are to be used for information only and the pretest values collected before the dosing period during acclimatization should be used for interpretation in the context of this study

^2^ P-values in bold denote significance

(−) Direct bilirubin was not analyzed for the pretest interval due to a protocol deviation

Similarly, clinical chemistry parameters were comparable between control and treated animals, with the exception of a treatment by time effect on albumin (*P* = 0.0254), direct bilirubin (*P* = 0.0717) and cholesterol (*P* = 0.0913) and a treatment effect on calcium (*P* = 0.0423).

All coagulation parameters were comparable between control and treated groups at all time points.

### Gross and microscopic evaluation

Two dogs had gastrointestinal tract lesions on gross examination: one male in Group 3 (6 mg/kg) had mild, red foci on the cecum and one female in Group 4 (10 mg/kg) had minimal, red mucosal discoloration of the duodenum. The gross lesions did neither correspond to any histological changes nor correlate to clinical findings in either dog. In addition, there were no relevant microscopic observations present in male or female dogs (summary data for histology are presented in Table 4).

**Table 4:** Summary data of histology

Tissue	Group 1 (sham)	Group 2 (2 mg/kg)	Group 3 (6 mg/kg)	Group 4 (10 mg/kg)
Observation, severity (localization)				
Adrenal glands				
Normal	8	8	8	8
Aorta				
Normal	8	8	8	8
Bone marrow				
Normal (femur)	8	8	8	8
Normal (rib)	8	8	8	8
Normal (sternum)	8	8	8	8
Bone				
Normal (femur)	7	7	8	8
Cartilage degeneration, minimal (femur)	1	1	0	0
Normal (rib)	8	8	8	8
Normal (sternum)	8	8	8	8
Brain				
Normal	7	8	8	8
Gliosis, minimal	1	0	0	0
Eyes, optic nerves				
Normal	8	8	8	8
Gallbladder				
Normal	8	8	8	8
Gastronintestinal tract				
Normal (esophagus)	8	8	8	8
Normal (stomach)	8	8	8	7
Focal mineralization, minimal (stomach, cardia)	0	0	0	1
Normal (duodenum)	8	8	8	8
Normal (ileum)	8	8	7	8
Mucosal atrophy, minimal (ileum)	0	0	1	0
Normal (jejunum)	8	8	8	8
Normal (cecum)	8	8	8	8
Normal (colon)	7	8	8	8
Cyst, mild (colon)	1	0	0	0
Normal (rectum)	8	8	8	8
Gonads				
Normal (ovaries)	4	4	4	4
Normal (testis)	2	4	3	3
Seminiferous tubules hypoplasia, minimal (testis)	2	0	1	1
Hypospermatogenesis, moderate (testis)	1	0	0	0
Heart				
Normal	7	8	8	8
Hematocyst, minimal (valvular)	1	0	0	0
Kidneys				
Normal	0	1	0	0
Lymphocytic infiltration, minimal	0	1	1	0
Tubular mineralization, minimal	8	7	8	8
Tubular regeneration, minimal	1	0	0	0
Liver				
Normal	8	7	7	4
Mononuclear infiltrate, minimal	0	1	1	4
Lung				
Normal	0	1	2	1
Hemorrhage, minimal	0	0	0	1
Alveolar histiocytosis, minimal or mild	6	6	4	5
Lymphocytic infiltration, minimal	6	3	3	3
Subacture inflammation, minimal	0	0	2	0
Lymph nodes				
Normal (mandibular)	8	8	8	8
Normal (mesenteric)	8	8	8	8
Normal (tracheobronchial)	8	8	8	8
Pancreas				
Normal	7	8	8	8
Lymphocytic infiltration, minimal	1	8	8	8
Pituitary				
Normal	6	8	6	7
Cyst, minimal or mild	2	0	2	1
Prostate				
Normal	4	4	1	4
Lymphocitic infiltration, minimal or mild	0	0	3	0
Salivary gland				
Normal	6	7	6	8
Lymphocytic infiltration, minimal	2	1	2	0
Skeletal muscle				
Normal	8	8	8	8
Skin				
Normal	8	8	8	8
Spinal cord (cervical, thoracic and lumbar)				
Normal	8	8	8	8
Spleen				
Normal	8	8	8	8
Thymus				
Normal	0	0	1	1
Cyst, minimal or mild	2	4	0	3
Lymphoid depletion, minimal to moderate	8	8	7	6
Thyroid				
Normal	8	8	8	8
Trachea				
Normal	8	8	8	8
Urinary bladder				
Normal	8	8	8	8
Uterus with cervix				
Normal	3	3	4	4
Cyst, mild	1	0	0	0
Pseudocyesis, mild	0	1	0	0
Vagina				
Normal	8	8	8	8

Data are the number of dogs with presence of normal histology or detected abnormalities

N = 8 per group (4 males and 4 females)

Summary data for organ weights are presented in Table 5. Mean ovarian weights were statistically significantly decreased in females in Group 3 and 4 (1.3 and 1.1 g, respectively) when compared to controls (1.9 g); however, there were no gross or histopathologic changes noted in the ovaries of any dog. Mean pooled spleen weights were statistically significantly higher in Group 2 and 4 (69 and 72 g, respectively) when compared to controls (51 g). Microscopically, all spleens were within normal limits. There were no statistically significant differences in individual male or female mean spleen weights when compared to controls. Mean pooled thyroid/parathyroid gland weight (relative to body weight) was statistically increased in Group 4 when compared to controls. The organ weight increase was minimal (15%) and there were no corresponding microscopic findings present. No other organ weight parameter showed statistical significance of interest.

**Table 5:** Summary of body (kg) and organ (g) weights; mean (SD), N = 8 unless stated

Organ	Group 1 (sham)	Group 2 (2 mg/kg)	Group 3 (6 mg/kg)	Group 4 (10 mg/kg)	P-value^1^ Treatment
Body weight	11.23 (2.43)	11.31 (2.28)	11.18 (1.69)	10.78 (1.68)	0.8599
Adrenal glands	1.05 (0.15)	1.10 (0.21)	1.09 (0.17)	1.09 (0.16)	0.9364
Brain	75.69 (6.15)	77.12 (4.93)	78.50 (7.30)	74.62 (4.03)	0.4629
Epididymides (*N* = 4)	3.70 (0.92)	3.82 (0.42)	4.35 (0.63)	3.90 (0.30)	0.4915
Heart	86.57 (19.57)	84.26 (13.63)	85.48 (18.00)	84.51 (17.31)	0.9714
Kidneys	54.25 (13.40)	50.60 (8.81)	49.37 (11.02)	53.59 (14.19)	0.5232
Liver	316.83 (58.45)	323.03 (37.76)	303.03 (55.02)	298.05 (60.58)	0.6478
Lung	90.49 (16.43)	87.50 (12.44)	87.09 (14.23)	90.07 (13.05)	0.7920
Ovaries (N = 4)	1.86 (0.51)	1.88 (0.22)	1.29 (0.30)	1.14 (0.41)	**0.0319**
Pituitary glands	0.07 (0.01)	0.07 (0.02)	0.07 (0.01)	0.08 (0.02)	0.3945
Salivary gland	5.45 (1.43)	5.36 (0.99)	5.69 (1.08)	5.14 (1.16)	0.8261
Spleen	50.93 (15.95)	69.04 (15.92)	52.13 (20.22)	71.59 (20.26)	**0.0131**
Testes (N = 4)	13.35 (4.02)	17.37 (3.19)	16.36 (4.48)	17.91 (2.86)	0.3470
Thymus gland	6.39 (3.49)	7.47 (3.34)	7.72 (3.62)	5.43 (1.77)	0.4936
Thyroid/parathyroid glands	0.87 (0.20)	0.88 (0.19)	0.89 (0.18)	1.00 (0.16)	**0.4457**

Footnote:

^1^ P-values in bold denote significance

Gross evaluation of tibial plateaus did not reveal any alterations in any animals. Histopathologic evaluation of medial and lateral tibial articular cartilage revealed minimal superficial collagen fibrillation, chondrocyte and proteoglycan loss in two male animals (one control and one in Group 3 [6 mg/kg]). This minimal, spontaneous change was seen in the inner half of the medial tibia (area not protected by meniscus) in both cases.

### Toxicokinetics

The Cmax and AUClast data following dosing on Days 1, 30, and 150 demonstrated consistent and adequate exposure of all treated dogs and reached comparable levels during study treatment (Table 6). There was one exception in Group 2 (2 mg/kg) where there was a marked increase (approximately 3–4 folds) in the mean Cmax and AUClast at Day 150. The mean Cmax were observed between 0.5 and 2 h post-dose for the three dose levels throughout the study duration. Blood concentrations were below the limit of quantitation (< 2 ng/mL) within 24 h post-dose in the Group 2 (2 mg/kg) and 3 (6 mg/kg) for all animals at all time points. For Group 4 (10 mg/kg), three animals on Day 30 and Day 150 had low but detectable blood levels (≤ 3 ng/mL) at 24 h post-dose, with a majority of the animals exhibiting blood concentrations below the limit of quantification. In general, the Cmax and AUClast showed a dose-related increase between the three dose groups and did not markedly change as a function of duration of administration, indicating absence of accumulation.

**Table 6:** Mean (CV%) pharmacokinetic parameters by group for robenacoxib administered orally once daily for 6 months at 3 dose levels

Group (Dose)	Day	Cmax (ng/mL)	AUClast (h x ng/mL)	Tmax (h)
2 (2 mg/kg)	1	1888 (55)	2091 (23)	1.03 (64)
	30	1384 (51)	2042 (32)	1.06 (58)
	150	5277 (45)	6629 (45)	0.94 (19)
3 (6 mg/kg)	1	4255 (51)	8626 (53)	1.28 (63)
	30	5940 (55)	9700 (24)	1.13 (51)
	150	4146 (64)	7602 (44)	0.81 (32)
4 (10 mg/kg)	1	6612 (44)	15,246 (35)	1.88 (19)
	30	5877 (39)	13,803 (22)	1.25 (52)
	150	4777 (36)	11,077 (32)	1.25 (52)

## Discussion

The objective of this study was to evaluate the safety of repeated oral administrations of robenacoxib in dogs and to identify adverse effects associated with overdoses and chronic administration. This pivotal study is the only 6-month target animal safety study performed with the commercial tablets of robenacoxib for dogs and was a mandatory component of the safety data package required for registration [20]. The dose levels and multiples thereof were chosen in accordance with the VICH GL43 [13] to identify a margin of safety.

Onsior™ tablets for dogs were first approved in Europe for chronic osteoarthritis at a dose of 1 mg/kg, with a 1–2 mg/kg range. The upper end of the dosage range (i.e. 2 mg/kg) was chosen for the 1X dosage and the classical 3X (6 mg/kg) and 5X (10 mg/kg) dosages were applied. Onsior™ tablets were later submitted in the United States for surgical indications at a dose of 2 mg/kg, with a 2–4 mg/kg range. For this indication, the dosages used in this study correspond to 0.5, 1.5 and 2.5 times the maximum targeted exposure. The investigational veterinary products were administered without feed in order to maximize systemic exposure since robenacoxib oral bioavailabilty is decreased (by approximately 25%) with co-administration with feed [19]. Lower exposure and a corresponding higher safety margin should occur if Onsior™ tablets are administered with feed.

To date, five NSAIDs of the coxib class are available for dogs (deracoxib, robenacoxib, cimicoxib, firocoxib and mavacoxib) and only robenacoxib is licensed for cats. To the author’s knowledge, there is no other disclosure of safety data generated with the other coxibs and with overdoses with the exception of deracoxib [21], which is also marketed by Elanco Animal Health. For cimicoxib, firocoxib and mavacoxib, only clinical safety data at the therapeutic doses are published [22–26].

No serious adverse events were reported during this study. All treated animals were in good general health throughout the study duration. It was therefore concluded that administration of robenacoxib was well tolerated at oral doses of 2 to 10 mg/kg once daily for up to 6 months and supports the safe use of Onsior™ tablets at the recommended doses.

Clinical findings (salivation and soft feces) were observed in all groups but more frequently in the treated animals. These observations were reported infrequently in a small number of dogs and commonly seen in dogs under laboratory conditions as well as in the normal dog population [27]. Thus, they were considered incidental. With the exception of minor neurological findings (decreased hopping and proprioception) in four treated animals and a minor ophthalmoscopic finding (single retinal fold) in one treated dog, at the end of the study, all animals appeared healthy. No explanation was provided to the observation of decreased hopping and causality to the treatment is unknown. Since the observation was made at the end of the study only, it is also unknown whether this condition was transient.

In the ECG, three ventricular premature complexes were noted on the terminal ECG of a dog in Group 2 (2 mg/kg). Rare ventricular premature complexes can be a normal variant in Beagles, occurring with a prevalence of 0.6% to 1.0% of control animals [28] during routine electrocardiography and 18.8% (males) to 26.1% (females) of normal animals in 18–24 h Holter studies [29]. The affected animal belonged to the low dose group, therefore the arrhythmia was unlikely to represent a treatment effect. The potential of COX-2 inhibitors to cause increased risks of cardiovascular diseases, including thromboembolic stroke and myocardial infarction, has been described after long-term use in humans with drugs like rofecoxib or celecoxib [30–33]. Since then, the cardiovascular toxicity of COX-2 inhibitors, as well as other non-selective NSAIDs, has been the subject of concerns and controversy in human medicine [34, 35]. However, to the author’s knowledge, cardiovascular effects during coxib therapy have not been reported in animals, including dogs [1]. It is unlikely that COX-2 selective inhibition poses a thrombogenic risk to dogs. Indeed, dogs do not develop atherosclerosis and are not pre-disposed to occlusion-induced arrhythmias. As compared to humans, they have many more branching vessels in the heart, hence have exceptional myocardial vascular collateralization capability. Moreover, published safety data on robenacoxib [8, 36] or other coxibs [10, 21, 25, 37, 38] did not highlight any cardiovascular risks in dogs.

A few hematology and clinical chemistry values (displayed in Tables 2 and 3) were statistically different from the controls in some treated groups. These differences were all of small magnitude and within the normal reference and/or pretest values. In addition, there were no dose dependency or correlative findings, thus considered incidental and neither toxicologically relevant nor related to treatment. The coagulation parameters and BMBTs were comparable between control and treated groups, despite an increase in BMBT in two treated dogs, that were marginally higher than normal (i.e. slightly above 5 min). No effect of treatment on urine or fecal analysis parameters was observed.

The weight variation in the spleens was most likely due to differences in contraction rates and removal of blood prior to being weighed. Although weight changes in the ovaries in the higher treatment groups were statistically significant, there were no histopathologic changes. The thyroid/parathyroid gland weight increase in Group 4 was minimal without any correlative histologic findings and was thus considered clinically irrelevant. No other effects on organ weights were observed.

There were no macroscopic changes at post-mortem examination and no histologic changes in the kidney or liver. Creatinine, urea concentration and urine analysis were normal and not different to controls in the treated dogs. The clinical pathology evaluations did not reveal abnormalities in the liver enzymes (alkaline phosphatase, alanine and aspartate aminotransferases). Overall, there were no signs of toxicity on kidney and liver. The gastrointestinal lesions on gross examination (cecal red foci and duodenum discoloration) were mild or minimal and not associated with any microscopic observations, changes in bodyweight, feed consumption, or serum total protein. The sporadic changes in albumin and feces consistency were considered as not relevant.

The histopathologic evaluation of tibial cartilage did not reveal any tissue damage. The minimal histologic change of the tibia was considered as non-treatment-related.

The observed inter-individual variability (in Group 2, coefficient of variations are ranging from 19 to 64%, 45–54% and 23–45% for Tmax, Cmax and AUClast, respectively) of the pharmacokinetic parameters is probably over-estimated in this study. The blood sampling schedule was established in order to monitor drug exposure, rather than document the full pharmacokinetic concentration-time profile of robenacoxib. Hence, a limited sampling schedule was selected not to interfere with the primary safety endpoints evaluation. Inter-individual varibility for Tmax is moderately elevated. More time points around the expected Tmax would be required for more accurate determination of the Tmax and Cmax. In addition, uncertainty on Cmax is closely correlated with uncertainty on AUC parameters.

In Group 2, while AUC and Cmax values from study day 1 to day 30 were comparable, results from day 30 to day 150 revealed a marked increase in mean Cmax and AUC. As all concentrations pre-dose and 24 h post-dose were below the limit of quantification, no accumulation occurred as expected by the short terminal half-life (approximately 1 h) [19] of robenacoxib. This increase was not observed in the other dose-groups, and thus considered as incidental. This is supported by the repeat-dose kinetics in a previous study of the same design but using non-final formulation [8], which showed no evidence of accumulation or changes in the pharmacokinetics of robenacoxib during repeated administration for 6 months. Altogether, there is no evidence of accumulation of robenacoxib with repeated treatment.

## Conclusion

This 6-month laboratory target animal safety study was required for the registration of the veterinary product Onsior™. Clinical findings in the study included salivation and soft feces observed both in control and treated animal, but more frequently in the treated groups. Sporadic abnormalities that were observed only in treated animals were: slight increased BMBTs in two animals, a single retinal change, minor neurological observations in four animals, ventricular premature complexes in one animal, minimal or mild gastrointestinal tract lesions on gross examination in two animals and mean ovarian weight decrease. The gross lesions or organ weight changes did not correspond to any histological changes and there were no other relevant microscopic observations. There were no indications of toxicity to the liver or kidney.

There were no serious adverse events during the study and all dogs were in good health until study completion. Robenacoxib also had no detrimental effects on cartilage or joints. No systemic accumulation of robenacoxib was observed. Altogether, this study supports the safe daily long-term use of Onsior™ tablets at the recommended labeled doses and a margin of safety up to 10 mg/kg when assessed in healthy beagle dogs.

## Abbreviations

ANOVA: Analysis of variance
APPT: Activated partial thromboplastin time
AUC: Area under the curve
BMBT: Buccal mucosal bleed times
Cmax: Maximum blood drug concentration
COX: Cyclooxygenase
ECG: Electrocardiogram
FDA-CVM: Food and Drug Administration’s Center for Veterinary Medicine
GLP: Good Laboratory Practice
GMP: Good Manufacturing practice
HPLC: High-Performance Liquid Chromatography
K2-EDTA: Ethylenediaminetetraacetic acid dipotassium
LC-MS/MS: Liquid chromatography tandem mass spectrometry
LLOQ: Lower limit of quantification
NSAID: Non-steroidal anti-inflammatory drug
RMANCOVA: Repeated measures analysis of covariance
Tmax: Time to maximum blood concentration following drug administration
VICH: Veterinary International Conference on Harmonization
